# Can the sustainable development goal 9 support an untreated early childhood caries elimination agenda?

**DOI:** 10.1186/s12903-024-04552-8

**Published:** 2024-07-11

**Authors:** Morẹ́nikẹ́ Oluwátóyìn Foláyan, Rosa Amalia, Arthur Kemoli, Ivy Guofang Sun, Duangporn Duangthip, Olunike Abodunrin, Jorma I. Virtanen, Ray M. Masumo, Ana Vukovic, Ola B. Al-Batayneh, Tshepiso Mfolo, Robert J. Schroth, Maha El Tantawi

**Affiliations:** 1 Early Childhood Caries Advocacy Group, Winnipeg, Canada; 2 Department of Child Dental Health, Obafemi Awolowo University, Ile-Ife, Nigeria; 3 Department of Preventive and Community Dentistry, Faculty of Dentistry, Universitas Gadjah Mada, Yogyakarta, Indonesia; 4 Department of Pediatric Dentistry and Orthodontics, University of Nairobi, Nairobi, Kenya; 5 Faculty of Dentistry, the University of Hong Kong, Hong Kong SAR, China; 6 Lagos State Health Management Agency, Lagos, Nigeria; 7 Department of Clinical Dentistry, University of Bergen, Bergen, Norway; 8 Department of Community Health and Nutrition, Tanzania Food and Nutrition Centre, Dar es Salaam, Tanzania; 9 Clinic for Pediatric and Preventive Dentistry, School of Dental Medicine, University of Belgrade, Belgrade, Serbia; 10 Department of Preventive Dentistry, Faculty of Dentistry, Jordan University of Science and Technology, Irbid, Jordan; 11 Department of Preventive and Restorative Dentistry, College of Dental Medicine, University of Sharjah, Sharjah, United Arab Emirates; 12 Department of Community Health, University of Pretoria, Pretoria, South Africa; 13 Dr. Gerald Niznick College of Dentistry, University of Manitoba, Winnipeg, Canada; 14 Department of Pediatric Dentistry and Dental Public Health, Faculty of Dentistry, Alexandria University, Alexandria, Egypt

**Keywords:** Sustainable development, Dental caries, Child, preschool, Health policy, Built environment, Industrial development, Innovation, Infrastructural development, Sustainable industries, Internet access

## Abstract

**Background:**

Early childhood caries (ECC) is a global public health challenge that requires innovation, infrastructure, and health system influences to bolster initiatives for its management and control. The aim of this scoping review was to investigate the published evidence on the association between ECC and the targets of the Sustainable Development Goal 9 (SDG9) concerned with industry, innovation, and infrastructure development.

**Methods:**

The scoping review followed the Preferred Reporting Items for Systematic Reviews and Meta-Analyses Extension for Scoping Reviews (PRISMA-ScR) guidelines. A search was conducted in PubMed, Web of Science, and Scopus between July and August 2023 using a search strategy related to the promotion of resilient infrastructure, sustainable industries, scientific research and innovation, access to the internet and ECC. Only English language publications were included. Studies that solely examined ECC without reference to the SDG9 targets were excluded.

**Results:**

The search yielded 933 studies for review. After screening for the eligibility and removing duplicates, 916 unique articles remained for further screening. However, none of the identified studies provided data on the association between resilient infrastructure, sustainable industries, scientific research and innovation, access to the internet and ECC.

**Conclusion:**

There were no primary studies that assessed the association between ECC and SDG9, even though the plausibility of a potential relationship exists. Future studies are needed to generate evidence on the link between ECC and SDG9 as this link may contribute to the reduction in the proportion of children with untreated ECC.

**Supplementary Information:**

The online version contains supplementary material available at 10.1186/s12903-024-04552-8.

## Introduction

Early childhood caries (ECC) is a significant global public health problem. It is characterized by the presence of decayed lesions in the primary teeth of children aged below six years [1]. It affects 514 million children worldwide [2], and poses considerable challenges to their oral health, overall well-being, and future development [3]. Underserved children face a high burden of ECC, as it ranks among the most prevalent unmet healthcare needs in this population [4, 5].

Untreated ECC detrimentally affects the growth, development, quality of life, and well-being of affected children [6–9], as well as the quality of life of their parents [9–11], with long-term health consequences [12]. It is also associated with poor physical and psychological development [13], sleeping difficulties, irritability, low self-esteem, decrease in school performance [9, 14], the risk of poor brain development [15] and substantial healthcare costs [16] associated with the use of general anesthesia or conscious sedation to treat severe cases [17–19].

Prioritizing the elimination of untreated ECC, is therefore crucial and essential [20–22]. However, achieving this goal requires a fresh approach that incorporates innovative strategies, the development of supportive infrastructure, and industry investment in new technologies and tools for its elimination. The United Nations’ Sustainable Development Goal 9 (SDG9) provides a platform to drive such a global agenda by aiming to establish resilient infrastructure, promote sustainable industrialization, and foster innovation [23]. The SDG9.1, SDG9.A and SDG9.B can enhance access to high-quality dental care through the creation of resilient infrastructure to promote economic and human growth, particularly in marginalized regions where the prevalence of ECC is highest [24]. In addition, the SDG9.2 and SDG9.3 can promote the development of a comprehensive approach to ECC management through access to the financial market [23]. Furthermore, SDG9.4 and SDG9.5 can facilitate advancements in oral health diagnostics, therapeutic approaches, while SDG9.C can facilitate access to informational materials, which are crucial for the control and prevention of ECC [23]. There is suggestive evidence that this link may be plausible as similar associations have been reported for adolescents [4, 25, 27], and the use of digital technology can influence the risk of ECC [28].

The elimination of ECC would, however, require critical new thinking. This is because, even though ECC can be prevented through simple yet effective measures like regular oral hygiene practices, healthy dietary habits, and early dental visits, its prevalence remains high [29, 30]. This is likely due to the complex and multifactorial nature of the disease, resulting from the intricate interplay of economic, and environmental factors that contribute to its onset and progression. In addition, the dependence of the child on parents for diet and oral hygiene contributes to the complexity of ECC management. Parental factors can increase the exposure of children to ECC behavioral risk factors such as poor oral hygiene practices, frequent consumption of sugary foods and beverages, and limited access to quality dental care [31]. In addition, biological factors such as enamel structure, the oral microbiome and genetics can influence a child’s susceptibility to ECC [4, 32, 33]. Furthermore, limited resources and cultural practices and beliefs can significantly impact oral health behaviors, making awareness campaigns and behavioral change challenging [34]. Poor awareness about preventive measures, especially among underserved populations, further exacerbates ECC [34].

**Fig. 1:**
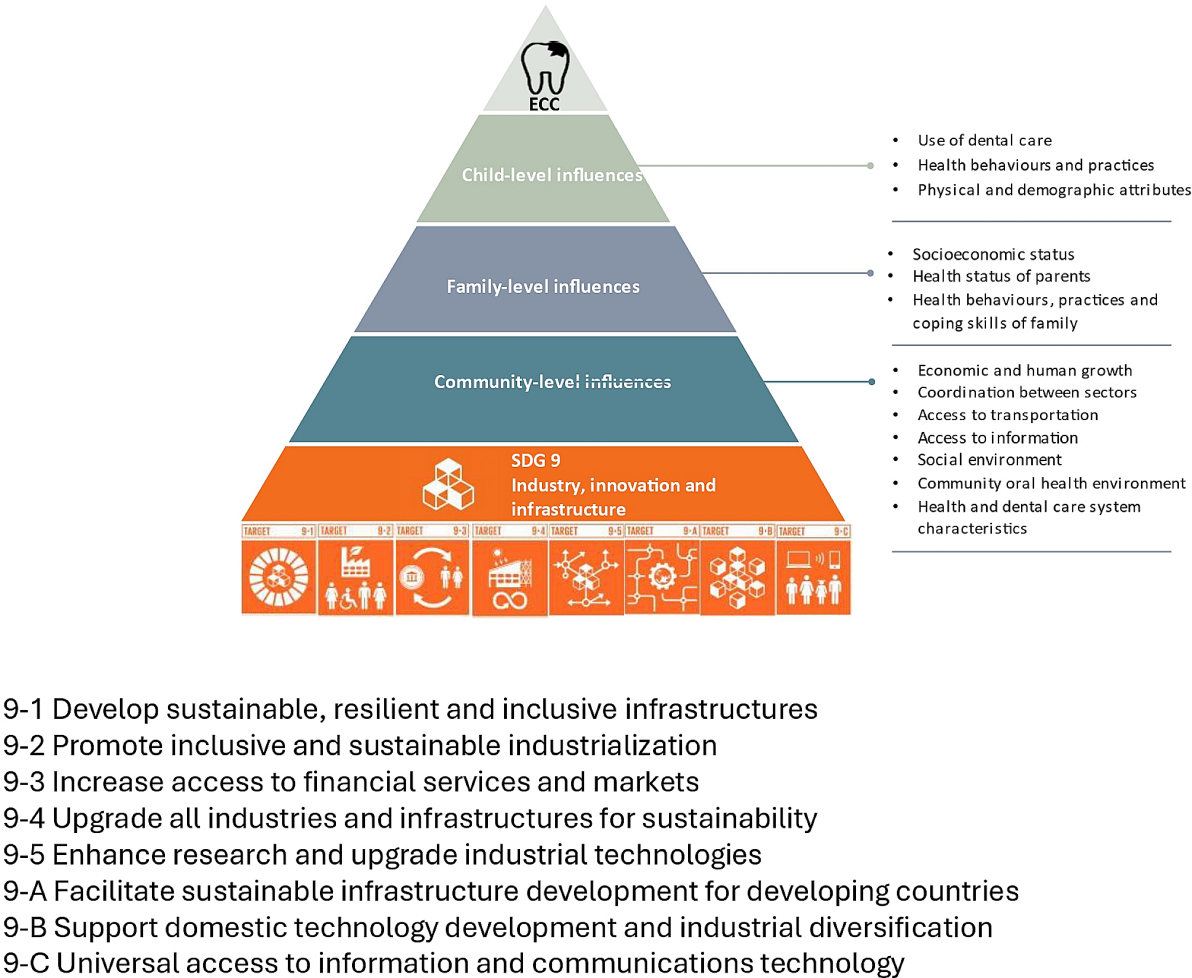
The conceptual framework of ECC and Industry, Innovation and Infrastructure (SDG9) as adapted based on the Fisher-Owens Model [35]

We, therefore, hypothesize, using the Fisher-Owens Model [35], that SDG9 has influence on community, family and individual level factors that affect the risk of ECC as illustrated in Fig. 1. The aim of this scoping review, therefore, was to map the published evidence on the association between ECC and the SDG9 to understand the link between the SDG9 and ECC, and to identify potential routes for ECC management using the SDG 9 framework.

## Methods

In this scoping review, we investigated the relationship between ECC, and the targets outlined in SDG9. SDG9 focuses on the establishment of resilient infrastructure, promotion of sustainable industrialization, and fostering innovation [23]. To ensure a systematic and transparent approach, we adhered to the Preferred Reporting Items for Systematic Reviews and Meta-Analyses Extension for Scoping Reviews (PRISMA-ScR) guidelines [36].

### Research question

The review was guided by the following question: What is the evidence on the link between promotion of sustainable industries, investment in scientific research and innovation, access to the internet and ECC?

## Search strategies

In August 2023, an initial search was conducted in three electronic databases: PubMed, Web of Science, and Scopus. The search strategy involved using relevant key terms listed in Appendix 1. The search terms were adjusted to suit the specific requirements of each database. Publications from the inception of the databases to August 19, 2023, were screened for eligibility.

### Inclusion criteria

This review only included publications written in English language and available until August 19, 2023. To be included, studies had to present findings on the association between industry, innovation, infrastructure, energy provision, scientific research, access to information and communications technology, and ECC.

### Exclusion criteria

Studies focusing on ECC only were excluded from this review. Ecological studies, review papers and non-primary quantitative research papers were also excluded from the full-text review screening and analysis.

### Article selection

The literature obtained from the database searches was exported to Zotero version 6, a reference management software. Duplicate publications were identified and removed using the “duplicate items” function. The screening process involved the evaluation of titles and abstracts by three independent reviewers (OA, MOF, MET), who followed the predetermined eligibility criteria for this scoping review. Full-text review of the remaining publications was then completed independently by two researchers (OA, MOF) and reference lists of potentially relevant publications were manually searched. Where consensus could not be reached, a third researcher (MET) was consulted. The summarized data was shared with experts for their review. For publications to be retained, there had to be consensus among all reviewers. No attempts were made to contact authors or institutions for additional sources.

### Role of the funding source

There was no external funding for the study. The study design selection, collection, analysis and interpretation as well as writing of the report were free from any form of influence. All authors had full access to the data in the study and shared the responsibility of the decision to submit for publication.

## Results

**Fig. 2:**
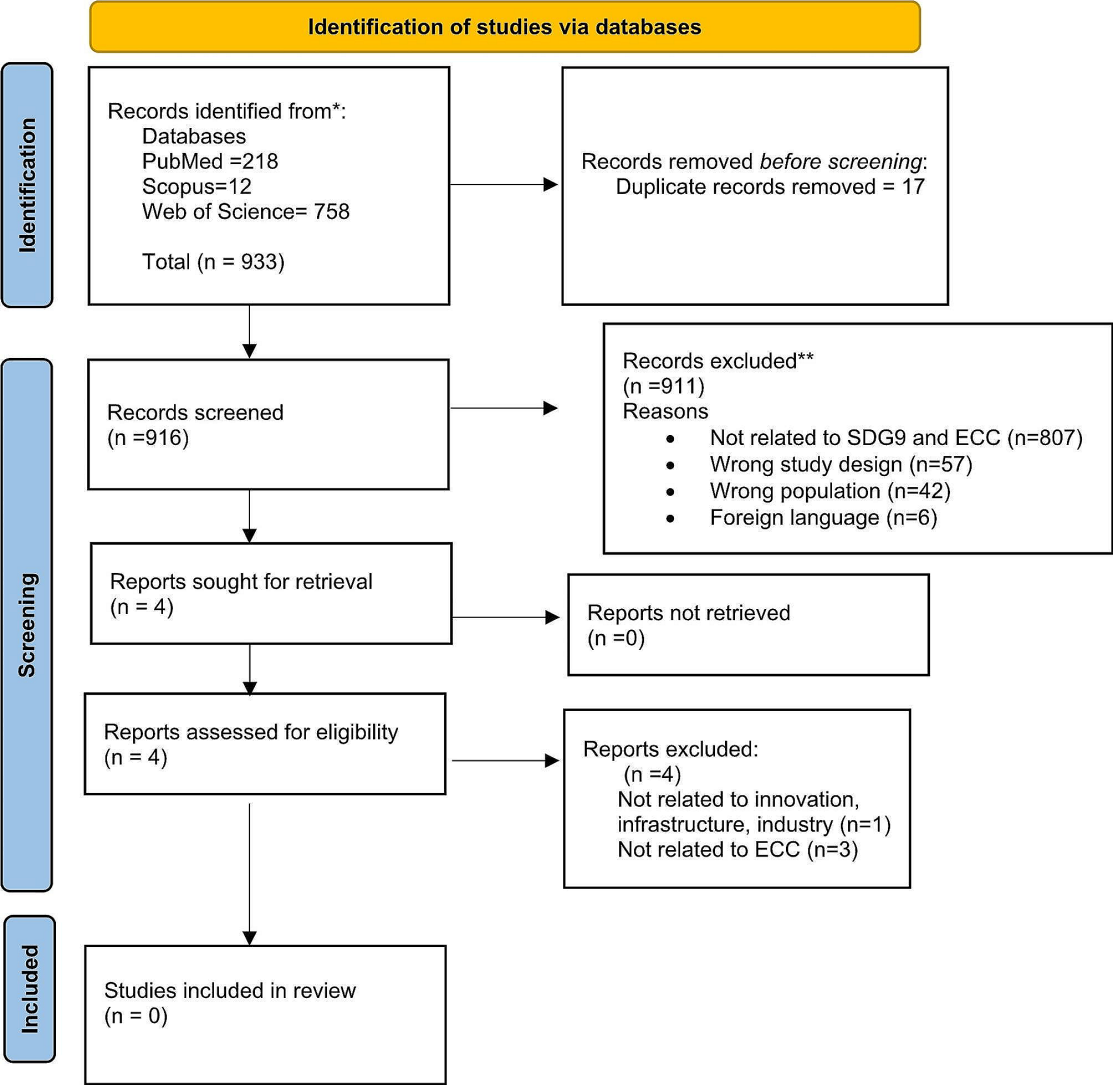
Flow diagram based on the Preferred Reporting Items for Systematic Reviews and Meta-Analyses 2020 flowchart template of the search and selected process

The search conducted in PubMed, Web of Science, and Scopus databases using the predefined search terms resulted in 933 articles. After screening for the eligibility and removing duplicates, 916 unique articles remained for further screening. However, none of the publications met the inclusion criteria. Figure 2 represents the flowchart for the study.

## Discussion

This scoping review’s objective was to map the published evidence on the association between ECC and the SDG9 to identify the implications and opportunities for addressing ECC within the broader context of infrastructure development, innovation, and industrialization. The result suggests that there is no scientific article exploring an association between SDG9 and ECC in the English literature. Nonetheless, SDG9 has the potential to positively impact oral health and mitigate the risk of ECC among young children. This influence may be through the interlinkages that SDG9 has with other SDGs, particularly SDG 11 (focusing on sustainable cities and communities), SDG 12 (emphasizing responsible consumption and production), and SDG8 (endeavouring to generate fresh prospects for innovation and employment in developing nations) [37].

We attributed the absence of published literature on the link between ECC and the SDG 9 to a limited amount of empirical research addressing the specific topic. It is crucial to keep in mind that the World Health Organization introduced the SDGs in 2015, and researchers worldwide are gradually recognizing the significance of incorporating SDG-related inquiries into their research. There is also increased interest and investment in global health aimed at addressing inequalities, a major driver of untreated ECC [29]. Eliminating these inequalities and their impact on oral health requires systematic and far-reaching efforts that use the intellectual and financial resources of multiple sectors and institutions through the active engagement of stakeholders within and outside of the oral health field [38]. As time passes, we can expect the body of evidence linking SDGs and ECC to expand.

In addition, the intersection of ECC (a public health issue) with SDG9, which focuses on infrastructure and industrialization, is quite unique. Research in this niche might be limited, as ECC is typically studied in the context of public health, dental care, and social determinants of health rather than in relation to industrial and infrastructural development. Yet, studying the intersection between public health, industrial, and infrastructural development is essential for creating environments that promote health, reduce disparities, and support sustainable and equitable growth. This integrated, upstream approach leads to more effective policies, innovations, and interventions that can improve health outcomes and enhance the quality of life for populations including that of children at risk of ECC.

One of the areas we anticipate major changes is in the use of communication technology to promote access to information on oral health. There are indications that internet users have better caries preventive behaviours, though individuals addicted to the internet have poorer oral health profiles [4, 25, 27]. In addition, digital applications can help parents and children acquire knowledge that improves oral hygiene, which may help with the control of ECC in the long run [28]. Social media is increasingly being used as a vehicle for early childhood oral health promotion [39]. In addition, the internet has enabled the adoption of teledentistry, which has significant implications for paediatric dental care [40, 41] including the management of ECC. The internet can also support public education about the definition, risk factors, and preventive care of ECC [42]. Studies are needed on the effectiveness of communication technology for the control of ECC. However, there are no mobile apps that adequately addressed dental caries prevention behaviors in children who are at risk of ECC [43].

In addition, there is substantial return on investment because of investing in research and innovation. Research and innovation create new jobs, including jobs in the healthcare sector [44, 45]. Each dollar spent creating new jobs in the health section results in an additional US$ 0.77 contribution to economic growth [39, 46, 47]. A cautious approximation of the returns from cardiovascular research in the United Kingdom suggests potential health gains of around 9% annually [48], while cancer research yielded returns of about 10% [49], and musculoskeletal research about 7% [50]. The ripple effect on the economy was estimated to range between 15% and 18%, and when coupled with the estimated monetized health benefits, this cumulative impact could reach as high as 25% [51]. Health research presents an opportunity for substantial return on investment [52]. However, we found no information showing the return on investing in oral health research and innovation. This information gap hinders efficient decision-making, accountability, resource allocation and resource prioritization necessary to drive oral diseases control.

Furthermore, enhancing the sustainability of the oral healthcare industry involves building a stronger oral health sector that effectively manages ECC within healthcare facilities. Although there is ample literature suggesting approaches to enhance sustainability in healthcare systems [53–55], there is currently no information on the practical strategies to implement these sustainability measures for oral health, despite the available opportunities. The effective use of artificial intelligence may enhance the sustainability of oral healthcare industry for providing oral health care for children [56] though the evidence for this is yet to evolve. These gaps in knowledge create opportunities for future empirical research exploring the links between SDG9, oral health and ECC.

Despite the absence of primary studies on the link between SDG9 and ECC, providing evidence on the links where they exist can contribute to enabling the oral health field to utilise the outcomes of the SDG9 to catalyse the elimination of untreated ECC and improve oral health outcomes in infants, toddlers, and preschoolers. The sustainable industrialization target of SDG9 can promote sustainable practices in manufacturing eco-friendly and cost-effective oral health products, including toothbrushes, toothpaste, and dental materials. By supporting research and innovation in oral health technologies, new interventions, and preventive measures for ECC can be developed.

However, investing in the SDG 9 will result in new job creation. A prior ecological study suggests that equitable access to job opportunities for women empowerment without due consideration and support for childcare may be associated with higher risk of ECC [57, 58]. Thus, it is important to develop monitoring indicators on the impact of the SDG9 on oral health, including the risk for ECC to reduce the likelihood of a negative impact [20]. For example, social and economic policy changes in New Zealand were associated with substantial and persistent widening of ethnic and socio-economic inequalities in ECC among five-year-old children with deterioration in the oral health of ethnic minority children in comparison to their European counterparts [59].

One of the limitations of this study was limiting the comprehensive search to three databases: PubMed, Web of Science, and Scopus. There could be relevant studies in other databases, grey literature, or unpublished work that were not captured. In addition, only English language publications up to August 2023 were included. Relevant studies published in other languages or after this date were not considered. Further, institutions were not contacted for additional sources making it possible that relevant unpublished data or ongoing studies were excluded.

In conclusion, though this scoping review found no publications in English on the association between the targets of SDG9 and ECC, these links are plausible. Studies are needed to generate evidence on these links to inform decision making and to create strategic actions to eliminate ECC. By bridging the gap between ECC and SDG9, we can further advance the global efforts to eradicate this preventable disease and improve the oral health outcomes of children.

### Electronic supplementary material

Below is the link to the electronic supplementary material.

Supplementary Material 1

## Data Availability

The datasets used and/or analysed for the study are publicly accessible.

## Abbreviations

ECC: Early Childhood Caries
PRISMA-ScR: Preferred Reporting Items for Systematic Reviews and Meta-Analyses Extension for Scoping Reviews guidelines
SDG: Sustainable Development Goal
