# Supplementary material for: Can the sustainable development goal 9 support an untreated early childhood caries elimination agenda?
Source: BMC Oral Health. 2024 Jul 11;24:776. doi: 10.1186/s12903-024-04552-8 (PMC11241917; doi:10.1186/s12903-024-04552-8)
Supplement: Supplementary file 1 — Supplementary Material 1 [file 12903_2024_4552_MOESM1_ESM.docx]

Appendix 1

Scopus

Bottom of Form

| History Count | Search Terms | Results |
| --- | --- | --- |
| 3 | #1 AND #2 | [12 document results](https://08105el9i-1105-y-https-www-scopus-com.mplbci.ekb.eg/search/history/results.uri?origin=searchhistory&shid=3) |
| 2 | TITLE-ABS-KEY ( caries )  OR  TITLE-ABS-KEY ( dental  AND  caries )  OR  TITLE-ABS-KEY ( dental  AND  decay )  OR  TITLE-ABS-KEY ( dental  AND  cavities )  OR  TITLE-ABS-KEY ( enamel  AND  demineralization )  OR  TITLE-ABS-KEY ( tooth  AND  demineralization )  OR  TITLE-ABS-KEY ( tooth  AND  cavities ) | [125,286 document results](https://08105el9i-1105-y-https-www-scopus-com.mplbci.ekb.eg/search/history/results.uri?origin=searchhistory&shid=2) |
| 1 | TITLE-ABS-KEY ( ( {industrial growth}  OR  {industrial diversification}  OR  {infrastructural development}  OR  {infrastructural investment}  OR  {infrastructure investment}  OR  {public infrastructure}  OR  {resilient infrastructure}  OR  {transborder infrastructure}  OR  {public infrastructures}  OR  {resilient infrastructures}  OR  {transborder infrastructures}  OR  ( {industrial emissions}  AND  mitigation )  OR  {industrial waste management}  OR  {industrial waste treatment}  OR  {traffic congestion}  OR  microenterprise*  OR  micro-enterprise*  OR  {small enterprise}  OR  {medium enterprise}  OR  {small enterprises}  OR  {medium enterprises}  OR  {small entrepreneur}  OR  {medium entrepreneur}  OR  {small entrepreneurs}  OR  {medium entrepreneurs}  OR  {value chain management}  OR  ( {broadband access}  AND  {developing countries} )  OR  {manufacturing innovation}  OR  {manufacturing investment}  OR  {sustainable transportation}  OR  {accessible transportation}  OR  {transportation services}  OR  {inclusive transportation}  OR  {R&D investment}  OR  {green product}  OR  {green products}  OR  {sustainable manufacturing}  OR  ( {cradle to cradle}  AND  industry )  OR  {closed loop supply chain}  OR  ( industrial  AND  innovation )  OR  {process innovation}  OR  {product innovation}  OR  {inclusive innovation} ) ) | [159,178 document results](https://08105el9i-1105-y-https-www-scopus-com.mplbci.ekb.eg/search/history/results.uri?origin=searchhistory&shid=1) |

WoS

- WOS.SCI: 1900 to 2023
- WOS.AHCI: 1975 to 2023
- WOS.BHCI: 2005 to 2023
- WOS.BSCI: 2005 to 2023
- WOS.ESCI: 2005 to 2023
- WOS.ISTP: 1990 to 2023
- WOS.SSCI: 1900 to 2023
- WOS.ISSHP: 1990 to 2023

| # | Search Query | Results |
| --- | --- | --- |
| 1 | ((((((((((((((((((((((((((((((((((((((((((TS=(industrial growth)) OR TS=(industrial diversification)) OR TS=(infrastructural development)) OR TS=(infrastructural investment)) OR TS=(infrastructure investment)) OR TS=(public infrastructure)) OR TS=(resilient infrastructure)) OR TS=(transborder infrastructure)) OR TS=(public infrastructures)) OR TS=(resilient infrastructures)) OR TS=(transborder infrastructures))) OR TS=(industrial waste management)) OR TS=(industrial waste treatment)) OR TS=(traffic congestion)) OR TS=(microenterprise* )) OR TS=(micro-enterprise*)) OR TS=(small enterprise)) OR TS=(medium enterprise)) OR TS=(small enterprises)) OR TS=(medium enterprises)) OR TS=(small entrepreneur)) OR TS=(medium entrepreneur)) OR TS=(small entrepreneurs)) OR TS=(medium entrepreneurs)) OR TS=(value chain management)) OR TS=(broadband access)) OR TS=(developing countries)) OR TS=(manufacturing innovation)) OR TS=(manufacturing investment)) OR TS=(sustainable transportation)) OR TS=(accessible transportation)) OR TS=(transportation services)) OR TS=(inclusive transportation)) OR TS=(R&D investment)) OR TS=(green product)) OR TS=(green products)) OR TS=(sustainable manufacturing)) OR TS=(closed loop supply chain)) OR TS=(industrial AND innovation )) OR TS=(process innovation)) OR TS=(product innovation)) OR TS=(inclusive innovation) | 946909 |
| 2 | ((((((TS=(caries)) OR TS=(dental caries)) OR TS=(dental decay)) OR TS=(dental cavities)) OR TS=(tooth cavities)) OR TS=(tooth deminerali?ation)) OR TS=(enamel deminerali?ation) | 67470 |
| 3 | #2 AND #1 | 758 |

Pubmed

| # | Query | Results |
| --- | --- | --- |
| 5 | #3 AND #4 | 218 |
| 4 | (((((("Dental Caries"[Mesh]) OR "Tooth Demineralization"[Mesh]) OR (caries[Text Word])) OR (dental decay[Text Word])) OR (dental cavities [Text Word])) OR (tooth cavities[Text Word])) OR (enamel demineralization[Text Word]) | 71,613 |
| 3 | #1 OR #2 | 88,455 |
| 2 | (((((((((((((((((((((((((((((((industrial growth[Text Word]) OR (industrial diversification[Text Word])) OR (infrastructural development[Text Word])) OR (infrastructural investment[Text Word])) OR (infrastructure investment[Text Word])) OR (public infrastructure[Text Word])) OR (resilient infrastructure[Text Word])) OR (transborder infrastructure[Text Word])) OR (industrial waste management[Text Word])) OR (industrial waste treatment[Text Word])) OR (traffic congestion[Text Word])) OR (microenterprise*[Text Word])) OR (micro-enterprise*[Text Word])) OR (small enterprise[Text Word])) OR (medium enterprise[Text Word])) OR (small entrepreneur[Text Word])) OR (medium entrepreneur[Text Word])) OR (value chain management[Text Word])) OR (broadband access[Text Word])) OR (manufacturing innovation[Text Word])) OR (manufacturing investment[Text Word])) OR (sustainable transportation[Text Word])) OR (accessible transportation[Text Word])) OR (transportation services[Text Word])) OR (inclusive transportation[Text Word])) OR (R&D investment[Text Word])) OR (green product[Text Word])) OR (sustainable manufacturing[Text Word])) OR (closed loop supply chain[Text Word])) OR (process innovation[Text Word])) OR (product innovation[Text Word])) OR (inclusive innovation[Text Word]) | 4,189 |
| 1 | (("Small Business"[Mesh]) OR "Developing Countries"[Mesh]) OR "Inventions"[Mesh] | 84,467 |

933 total

Duplicates removed, remaining= 916
